# The Host Genetic Diversity in Malaria Infection

**DOI:** 10.1155/2012/940616

**Published:** 2012-12-13

**Authors:** Vitor R. R. de Mendonça, Marilda Souza Goncalves, Manoel Barral-Netto

**Affiliations:** ^1^Laboratório Integrado de Microbiologia e Imunorregulação (LIMI), CPqGM, FIOCRUZ, Rua Waldemar Falcão 121, Candeal, 40296-710 Salvador, BA, Brazil; ^2^Faculdade de Medicina, Universidade Federal da Bahia, 40296-710 Salvador, BA, Brazil; ^3^Faculdade de Farmácia, Universidade Federal da Bahia, 40296-710 Salvador, BA, Brazil; ^4^Instituto de Ciência e Tecnologia do Sangue, Campinas, SP, Brazil; ^5^Instituto de Investigação em Imunologia, Instituto Nacional de Ciência e Tecnologia, São Paulo, SP, Brazil

## Abstract

Populations exposed to *Plasmodium* infection develop genetic mechanisms of protection against severe disease. The clinical manifestation of malaria results primarily from the lysis of infected erythrocytes and subsequent immune and inflammatory responses. Herein, we review the genetic alterations associated with erythrocytes or mediators of the immune system, which might influence malaria outcome. Moreover, polymorphisms in genes related to molecules involved in mechanisms of cytoadherence and their influence on malaria pathology are also discussed. The results of some studies have suggested that the combinatorial effects of a set of genetic factors in the erythrocyte-immunology pathway might be relevant to host resistance or susceptibility against *Plasmodium* infection. However, these results must be interpreted with caution because of the differences observed in the functionality and frequency of polymorphisms within different populations. With the recent advances in molecular biology techniques, more robust studies with reliable data have been reported, and the results of these studies have identified individual genetic factors for consideration in preventing severe disease and the individual response to treatment.

## 1. Introduction

Malaria is one of the most important and prevalent infectious diseases in the world. The World Health Organization (WHO) estimated 225 million malaria cases worldwide with 781,000 deaths due to *Plasmodium* infection per year [1]. Four species of *Plasmodium* (*P. falciparum*, *P. vivax*, *P. malariae*, and *P. ovale*) are responsible for almost all human infections [2]. 

Malaria has been associated to gene selective pressure in the human genome, and it has been associated as an evolutionary force of some genetic diseases, such as sickle cell disease (SCD), thalassemia, glucose-6-phosphate dehydrogenase (G6PD) deficiency, and other red blood cell (RBC) genetic anemia with Mendelian inheritance. Haldane (1949) suggested a “balanced polymorphism” where the hemoglobin S (HbS) homozygote disadvantage is recompensed through the resistance of the heterozygote (HbAS) in regions where malaria is endemic [3]. Reports associating several genetic disorders with malaria susceptibility or resistance are on the rise, and studies of heritability indicate that approximately 25% of the risk for severe malaria progression is determined through human genetic factors [4]. 

Genetic epidemiology may help in pointing out major molecular pathways of some infectious diseases, such as malaria, which involve a robust immune and inflammatory response and the participation of erythrocytes and other blood cells in its pathogenesis. The aim of this paper is to review the major genetic alterations in the human host associated with the clinical spectrum of malaria infection and disease development. We specifically address the areas of inherited disorders in red blood cells (RBC) and mutations in the genes of key molecules during the immune response that confer an increase of susceptibility or resistance against malaria. The multiplication of *Plasmodium* inside the RBC and its subsequent rupture have been implicated in several phenomena present in the malarial syndrome. A protective effect against malaria infection has been associated with genetic disorders involving the RBC, such as cytoskeleton disorders, surface antigen gene mutations, enzymatic machinery deficiencies, or hemoglobin alterations [5]. The immune response is critical for controlling* Plasmodium* infection, and the balance between proinflammatory (Th1-type) and anti-inflammatory (Th2-type) cytokines has been implicated in both the control of parasite multiplication and the development of symptoms. The genetic background of the affected individual might also influence cytokine expression and disease outcomes [6, 7]. Notably, the frequency of genetic alterations differs depending on the population origin and structure, and some mutations might differentially influence the disease outcome in different patterns. 

Understanding the genetic alterations involving RBC disorders and the immune response might provide insight into the development of new strategies for host-genotype treatment and/or the prevention of malaria.

## 2. Inherited Disorders of Red Blood Cells and Malaria 

### 2.1. Membrane and Enzymatic Disorders of Erythrocytes

Several membrane-inherited disorders of RBC provide additional information concerning the pathogenesis of malaria. Hereditary spherocytosis is a disorder characterized by the loss of membrane lipid surface. This common hemolytic anemia also reflects ineffective integral protein interactions and is associated with lower parasitemia [8]. Other RBC membrane inherited disorders include hereditary ovalocytosis, elliptocytosis, pyropoikilocytosis, and acanthocytosis. Elliptocytosis has been associated to resistance against invasion by *P. falciparum* in humans and *P. knowlesi* in experimental models [9]. However, ovalocytosis is a RBC-inherited cytoskeleton disorder most commonly associated with malaria. A particular type of southeast Asian ovalocytosis (SAO), also known as Melanesian elliptocytosis or stomatocytic elliptocytosis, is characterized by an inherited dominant trait related to heterozygosity for a 27-pair deletion in the gene encoding protein band 3 (*SLC4A1Δ27*) in the erythrocyte membrane [10]. Although SAO homozygosity has been associated with embryo mortality, its heterozygosity is associated with the absence of clinical symptoms and a lack of hemolysis. Ovalocytes are characterized as rigid and more resistant to changes in the shape as a result of low osmotic fragility and the low expression of several RBC antigens [11]. SAO ovalocytes characteristics have been associated with resistance to malaria infection, particularly against *P. falciparum* merozoites invasion [11]. Patients with elliptocytosis exhibit a similar degree of parasitemia independent of disease severity [11–13]. 

The Duffy, also called Duffy antigen/chemokine receptor (DARC), Fy glycoprotein, or CD234, is a RBC antigen encoded by the *DARC* human gene that is considered to be a nonspecific receptor for several chemokines. The *P. vivax* merozoite uses the Fy antigen to invade RBC [14]. The *Fy* antigen possesses two distinct alleles known as *Fya* and *Fyb*, which result from a single point mutation in codon 42 (rs2814778) that results in a glycine to asparagine substitution within the protein. Another polymorphism (−33T>C, no rs designation available) in the promoter region of the *DARC* gene ablates DARC expression on the surface of erythrocytes. Erythrocytes expressing *Fya* exhibited 41–50% lower binding to *P. vivax* compared with *Fyb*, and individuals with the Fya+b-phenotype showed a 30–80% lower risk of developing clinical symptoms of vivax malaria [15]. The RBC of individuals with the *Fy-33* genotype are not susceptible to *P. vivax* merozoites invasion and are refractory to the erythrocytic stage of the disease. However, the hepatic malaria stage has been observed in these individuals, making them reservoirs for the disease [14]. With regard to *P. falciparum* infection, more than one receptor on the surface of RBC is responsible for merozoite infection, which include glycophorin A, B, and C (GPA, GPB, and GPC), protein band 3 and others (Y receptors, E, Z, and X), whose molecular identity has not yet been determined [16]. The genetic polymorphisms in the *band 3* or *G*Υ*PC* gene are highly prevalent in malaria endemic areas (Papua New Guinea) and confer resistance to severe diseases [17]. Polymorphisms identified in other receptors genes, such as *G*Υ*PA* and *G*Υ*PB*, have been shown to confer only partial protection against the *Plasmodium* invasion of RBC [18, 19]. Furthermore, in the Brazilian Amazon, an SNP in the *G*Υ*PB* receptor gene (rs7683365) was associated with host susceptibility to *P. falciparum* infection [17].

The G6PD deficiency and low levels of pyruvate kinase are the most prevalent genetic alterations in RBC that can influence malaria outcomes. G6PD is a metabolic enzyme that catalyzes the first reaction in the pentose phosphate pathway, providing energy for the RBC in the form of nicotinamide adenine dinucleotide phosphate (NADPH). NADPH enables RBC to counterbalance oxidative stress through oxidant agents [20]. The *G6PD* gene is located on the X chromosome and is therefore more prevalent in men [21]. The G6PD deficiency is the most common cause of hereditary hemolytic anemia and is more prone to oxidative stress from the decreased production of NADPH. The clinical picture of G6PD deficiency involves different degrees of disease severity, which might include hemolytic anemia, neonatal hyperbilirubinemia, and asymptomatic cases [22]. Approximately 400 million people living in tropical and sub-tropical areas exhibit a G6PD deficiency, with a high diversity of variants, including the common *G6PD B* (wild type), *G6PD A* (nondeficient type), and *G6PD A- *(African deficient type) [23]. It has been suggested that G6PD deficient RBC might reduce intracellular parasite growth [24]. Moreover, studies have shown that infected erythrocytes deficient of G6PD were more phagocytosed by monocytes, which might be associated with the reduction of the parasitic load of the disease [24, 25]. Another enzyme associated with energy production is pyruvate kinase (PK), which is also an important factor in the susceptibility to malaria, and its deficiency has been associated with the reduced survival and increased phagocytosis of parasite-infected erythrocytes [26]. PK deficiency is the second most common cause of hereditary nonspherocytic hemolytic anemia in humans [27]. PK catalyzes the rate-limiting step of glycolysis, and the energy for erythrocytes is derived from glycolysis, as RBC lack mitochondria. The *PK* gene is highly pleomorphic and includes 59 SNPs and several loss-of-function variants that might be associated with decreased resistance to malaria [27].

### 2.2. Hemoglobin Alterations

Hemoglobin (Hb), the main compound of erythrocytes, is a tetrameric protein that consists of two pairs of unlike globin chains; each globin chain is associated with one prosthetic group, called a heme group. Hemoglobinopathies are inherited disorders of Hb that can be classified into two major groups. The first group involves structural alterations or variants of Hb, such as HbS, HbC, and HbE; the second group of hemoglobinopathies is classified as synthesis defects of Hb and has been associated with a decrease or absence of globin chain synthesis, with the most common alteration related to the alpha and beta-globin chains (alpha- and beta-thalassemia, resp.).

The alpha-thalassemia (alpha-thal) is the most common genetic disorder in the human population and is caused by the decreased or absent synthesis of the alpha globin chain due to the deletion or nondeletion mutation of one or both *alpha-globin* genes (*HBA1* and *HBA2*), located on chromosome 16 [28, 29]. A 3.7-kilobase (Kb) deletion determines the most common form of alpha-thal, also called alpha^+^-thal, and there is unique potential for a rightward crossover between the *HBA* genes. One study reported that alpha^+^-thal increased the incidence of mild malaria [5]. However, several subsequent studies reported that alpha^+^-thal was associated with a reduced risk of uncomplicated malaria episodes [30] or a protective effect against severe forms of malaria [31–35]. 

Beta-thalassemia is characterized by the decreased synthesis of the beta-globin chain through a genetic alteration in the *beta-globin* gene (*HBB*) of the human *globin* gene cluster located on chromosome 11 [36]. The heterozygote of this inherited trait is associated with a mild anemia and an ineffective erythropoiesis, while the homozygote mutant is associated with severe anemia and the risk of early death. The beta-thalassemia trait is associated with a relative resistance against *P. falciparum* malaria [37] and protection against severe malaria forms [38]. 

The presence of a single nucleotide mutation (rs334) in the *beta-globin* gene (*HBB*) is associated with a structural modification of the beta polypeptide chain Glu6Val (HbS), resulting in the variant HbS, which can be found in the asymptomatic heterozygous state. This variant is commonly known as the sickle cell trait (HbAS). In 1978, Friedman suggested that the mechanism of resistance against *Plasmodium* of RBC with HbS might be solely due to intraerythrocytic conditions [39]. HbS polymerizes under deoxygenate conditions, and parasites become severely affected; HbS RBC become sickled, with the increased phagocytosis of infected erythrocytes [40, 41]. The presence of HbS in severe malaria patients is associated with less hemolysis and reduced levels of free heme [42, 43]. Many studies have described an association between the heterozygote HbAS and protection against malaria, with more than 90% protection against severe forms [44, 45]. A large genome-wide association (GWA) analysis of severe malaria cases in four different ethnic groups in Gambia has confirmed the role of HbS variant in resistance to malaria [46]. However, this study did not find new genetic associations as a result of the need for population-specific data on genome sequence variation. Therefore, it is difficult to design effective multicenter replication studies without information about sequence variation and haplotype structure in those African populations [46]. Moreover, in another GWA study carried out in Ghana, besides confirmation of previous reports on protective effects of HbS and blood group O, two novel resistance loci were described for severe malaria [47]. One of the loci was identified on chromosome 1q32 within the *ATP2B4* gene, which encodes the main calcium pump of erythrocytes; and the other locus was indicated by an SNP on chromosome 16q22.2, possibly linked to a gene encoding the tight-junction MARVELD3, which may have a role in microvascular damage caused by endothelial adherence of parasitized erythrocytes [47]. 

Hemoglobin C (HbC) occurs from a point mutation leading to a Glu6Lys substitution at the sixth position in the beta-globin polypeptide chain (rs33930165) [48], and hemoglobin E (HbE) results from a substitution of Glu26Lys at position 26 in the beta-globin polypeptide chain (rs33950507) [49]. HbC homozygotic individuals exhibit mild hemolysis and splenomegaly, while the heterozygotic state is asymptomatic [50]. The RBC from HbE homozygous individuals is microcytic, with low hemoglobin concentrations, which reduces the possibility of merozoite invasion and impairs parasite growth within the variant RBC [51]. The presence of both protective factors, HbC and HbE, has been associated with a lower risk of developing severe forms of malaria [48, 49, 51–54]. 

Genetic alterations in erythrocytes were probably the first to be discovered as a result of the evolutionary pressure of malaria in the human genome. Mutations affecting several protection mechanisms related to RBC have been described for HbS [55]. Furthermore, several polymorphisms within the membrane cytoskeleton, surface antigen, and enzymatic machinery and other hemoglobin alterations within RBC influence the susceptibility and resistance against malaria.

### 2.3. Systemic Regulations of Heme

During the erythrocytic stage of malaria, merozoites multiply inside RBC, which result in the rupture of this structure and subsequent release of free Hb into the circulating blood. In the presence of reactive oxygen species (ROS), Hb releases its heme prosthetic group. In addition, the *Plasmodium* is responsible for the degradation of 60% to 80% of the total Hb [56]. Hb degradation contributes to heme release and ROS generation, which are harmful to both erythrocytic schizonts and the host [57]. Free heme is harmful to cells and tissues and can induce oxidative stress, inflammation, cytotoxicity [58], and cell death [59]. The *Plasmodium* has developed a series of protective mechanisms against the deleterious effects of free heme through the polymerization of heme in hemozoin, a malarial pigment that counteracts the pro-oxidative effects of iron (Fe) present in protoporphyrin IX [56]. The host also displays mechanisms of protection against free Hb cytotoxicity. Under homeostatic conditions, free Hb is released from the intravascular lysis of RBC and rapidly binds to haptoglobin (Hp). The CD163 receptor, expressed on monocytes/macrophages in the red pulp of the spleen, recognizes and internalizes the Hb/Hp complex [60]. Following internalization of the Hb/Hp complex, heme is degraded through the enzymatic action of heme-oxygenase (HO-1), producing biliverdin, iron, and carbon monoxide (CO). The beta chain (*β*) of Hp is approximately 40 kDa, and the alpha 1 chain (*α*1), which is synthesized by the allele variant *Hp1*, is approximately 8.86 kDa and is subdivided into *1S* and *1F*; the alpha 2 chain (*α*2), which is synthesized by the allele variant *Hp2*, is approximately 17.3 kDa [61]. These alleles have different affinities for free Hb (*Hp1.1* >* Hp1.2* >* Hp2.2*) and for CD163 (*Hp2.2* >* Hp1.2 > Hp1.1*) [60]. The presence of the *Hp2.2* genotype has been associated with increased iron redox activity and oxidative stress compared with the *Hp1.1* genotype [62, 63]. Furthermore, monocytes can internalize the Hb/Hp2.2 complex, but not Hb/Hp1, and stimulate the release of proinflammatory cytokines [64]. Thus, the *Hp2.2* has been associated with an increased susceptibility to various inflammatory conditions, including malaria. We have recently reported that subjects with the *Hp2.2* genotype display a higher risk of developing symptomatic mild malaria (as opposed to asymptomatic) upon *Plasmodium* infection [65].

The induction of HO-1 in experimental models of malaria has been associated with increased resistance to malaria as a result of HO-1 in controlling heme-induced nonspecific tissue damage and inflammation [55, 66]. There is an (GT)n repeat polymorphism microsatellite in the promoter of the *HMOX1* gene, which is associated with the increased or decreased synthesis of HO-1 in response to different stimuli [67]. Individuals with lower (GT)n dinucleotide repeats have a higher expression of HO-1, while higher (GT)n dinucleotide repetition is associated with the decreased synthesis of the protein [67]. This polymorphism has been described in several chronic degenerative diseases [68–73], but its role in malaria is controversial. The results of some studies have shown that the presence of lower (GT)n repeats in *HMOX1* gene is associated with a greater chance of developing severe malaria, suggesting that the increased expression of HO-1 is deleterious for human malaria [74, 75]. We have shown that subjects with the long form (≥30 GT repeats) of the *HMOX1* gene polymorphism have greater susceptibility to developing malaria and higher inflammatory scores than individuals with the short form [65]. However, studies in mice have shown that HO-1 is highly beneficial for malaria by conferring host tolerance to *Plasmodium* infection. Sickle Hb induces the expression of HO-1, which leads to CO production. CO binds with high affinity to free Hb and prevents the release of heme from hemoglobin, which reduces systemic levels of deleterious free heme [55, 66]. Other *HMOX1* gene polymorphisms have been described, which correspond to the single nucleotide polymorphisms −1135G>A (no rs designation available) and −413A>TG (no rs designation available). However, only the last SNP seems to have functional importance [67].

Heme reduces the production of prostaglandin E2 (PGE2) and TGF-*β* from mononuclear cells through superoxide dismutase-1 (SOD-1), an enzyme responsible for the detoxification of harmful superoxide [76]. SOD-1 is a powerful predictor of malaria severity in individuals infected with *P. vivax* with higher sensitivity and sensibility than TNF-*α* levels [77], confirming the importance of this enzyme in malaria pathogenesis. The preliminary data from our group showed an association between several SNPs in the *SOD-1* gene and different expressions of this enzyme in subjects with malaria. Furthermore, those *SOD-1* SNPs were associated with malaria symptoms in individuals infected with *P. vivax*, indicating that the genetic predisposition of the individual might alter the response of these subjects against ROS.

## 3. Immune Response

### 3.1. Toll-Like Receptors (TLR)

Toll-like receptors are a family of transmembrane proteins present in monocytes, macrophages, and dendritic cells, which play a key role in the innate immune response. TLR recognize pathogen associated molecular patterns (PAMPs) through extracellular receptor modules and initiate the inflammatory cascade through the transcription of inflammatory cytokines, type 1 interferon, and chemokines through NF-kB or interferon regulatory factor dependent pathways [78, 79]. Furthermore, the stimulation of TLRs also leads to dendritic cell maturation and the induction of the adaptive immune response [80]. Each TLR has a unique pattern of expression, intracellular localization, and signaling pathway, resulting in different immune responses [81, 82]. The intracellular signaling of TLR is mediated through at least five different adapter proteins, including toll-interleukin 1 receptor domain containing adaptor protein (TIRAP), myeloid differentiation primary response gene 88 (MyD88), and toll-like receptor adaptor molecule 1 (TRIF) [83]. TLR1, 2, 4, 5, 6, and 10 are found on the extracellular surface of cells, whereas TLR3, 7, 8, and 9, each of which is a nucleic acid sensor, are located within the endoplasmic reticulum and cytoplasmic vesicles [84]. In the context of malaria infection, TLR2 and TLR4 have been reported to recognize *P. falciparum* glycosylphosphatidylinositol (GPI), while TLR9 has been reported to recognize *Plasmodium* DNA or the hemozoin pigment [85–87]. 

Some common SNPs in *TLR* genes are functionally important and affect the recognition of ligands and intracellular signaling. These SNPs have been associated with numerous infectious and parasitic diseases [88]. Several studies have associated *TLR* gene polymorphisms with clinical malaria and parasitemia levels. Two polymorphisms have been described in the 5′ untranslated region (UTR) of the *TLR2* gene, a 22-base pair deletion in the first untranslated exon (Δ22), and an (GT)n dinucleotide repeat in the second intron [89]. Both polymorphisms, the deletion and shorter (GT)n repeats, are associated with reduced TLR2 reporter activity and TLR2 expression [90]. However, only the Δ22 heterozygous genotype was associated with protection from cerebral malaria [91]. Other SNPs of *TLR2* (Arg677Trp, no rs designation available and Arg753Gln, rs5743708) were not identified in the *Plasmodium*-infected population [92]. In the case of the *TLR4* gene, the more frequent genetic alterations studied were two nonsynonymous cosegregating SNPs (Asp299Gly, rs4986790 and Thr399Ile, rs4986791) that modify the ligand-binding site of the receptor [93]. The *TLR4* Asp299Gly was associated with an increased risk of maternal anemia and infant low-birth weight in pregnant women with malaria [94]. In addition, the risk of severe malaria in children was increased 1.5-fold in the presence of *TLR4* Asp299Gly and 2.6-fold with *TLR4* Thr399Ile [92]. 

The *TLR9* gene has been associated with the pathogenesis of severe malaria in humans and experimental models. Studies have demonstrated that TLR9-deficient mice survived more during cerebral malaria and that the antagonist-mediated TLR9 inhibition conferred protection against cerebral malaria in mice [95, 96]. A human study analyzing the (rs187084, −1486C>T) polymorphism in the promoter region of *TLR9* gene showed an increased risk of low birth weight among infants from pregnant women with malaria, whereas increased parasitemia was observed in adults with mild malaria [84, 94]. However, in a large family and population-based association study, Malawi and Gambia showed that the effects of the four most common SNPs in the *TLR9* gene, rs187084 (−1486C>T), rs5743836 (1237C>T), rs352139 (1174G>A), and rs352140 (2848G>A), were not associated with severe malaria [97]. Smaller population studies in Brazil, Iran, and Ghana showed associations between these polymorphisms and mild clinical malaria in their respective populations, raising the possibility that the *TLR9* gene polymorphisms might be associated with a milder form of the disease [84, 98, 99]. The TIRAP/Mal interaction with tumor necrosis factor receptor-associated factor 6 (TRAF6) is responsible for mediating the downstream signaling of TLR2 and TLR4 to induce a proinflammatory response [100]. An SNP mutation in the *TIRAP* gene (rs8177374; S180L) has been associated with both protection against malaria [101] and susceptibility to the development of mild malaria [97], but the association of the *TIRAP* gene polymorphism with malaria remains controversial.

The importance of TLR in malaria infection has been recently described, particularly with regard to TLR2, 4, and 9. Genetic alterations in *TLR* and their signaling pathways remain controversial. Thus far, no conclusive evidence of polymorphisms in these receptors that might influence the disease outcome and effect host-genotype treatment have been identified.

### 3.2. Cytokines

Malaria infection is marked by changes in cytokine expression resulting from individual immune responses. Proinflammatory Th1-type cytokines (IL-1, IL-6, IL-8, IL-12, IFN-gamma, and TNF-*α*) are critical for controlling the erythrocytic and hepatic stages of *Plasmodium* infection [6, 7], but the excessive production of these cytokines might also contribute to disease manifestations and/or tissue damage, such as the brain in cases of cerebral malaria. It has also been suggested that anti-inflammatory Th2-type cytokines (IL-4, IL-10, and TGF-beta) downregulate Th1-type cytokines and the proinflammatory response, thereby preventing subjects from severe forms of malaria [102]. Furthermore, the new concept of tolerance against diseases has demonstrated protection against malaria. Unlike resistance, tolerance does not affect the pathogen burden but reduces tissue damage and other pathological effects of disease caused by the pathogen or immune response [103]. This tolerance might be due to the genetic profile of the affected individual, and HbS and HO-1 expressions are the most described mechanisms in malaria [55, 66, 104].

TNF-*α* is a proinflammatory cytokine that has attracted particular interest because of its ambiguous activity in host defense and pathogenesis of cerebral malaria and other serious complications [105]. High concentrations of TNF-*α* are related to the pathogenesis of symptoms associated with malaria, such as fever, and severe forms of infection, such as cerebral malaria [106, 107]. However, TNF-*α* has also been associated with the presence of potent antiparasitic activity, and persistent high levels of the cytokine lead to a rapid improvement in fever and a reduction in parasitemia [108, 109]. Genetic alterations in the *TNF* gene have been described in several studies with different populations in the world and sometimes with contradictory results. Population differences in susceptibility or resistance to malaria according to *TNF* SNPs may be a result of diverse evolutionary pressure between ethnicities, as well as different parasite strains and incidence of severe forms of disease. In Gambia, the SNPs *TNF *−308G>A (rs1800629) and *TNF *−238G>A (rs361525) were associated with an increased risk of cerebral malaria and severe malarial anemia, respectively [110, 111]. Studies in Gabon associated the *TNF *−308G>A polymorphism with a shorter interval to malaria reinfection and the *TNF *−238G>A polymorphism with protection against mild symptomatic malaria [23, 112]. In Sri Lanka, the *TNF *−308A allele was associated with severe malaria and other infections [113]. In another study in Mynamar, the *TNFPD* allele haplotype (−238G; −308G; −857T, rs1799724; −1031T, rs1799964) was associated with increased susceptibility to cerebral malaria because the transcription factor OCT-1 binds to *TNF *−857T in the *TNFPD* allele but not to *TNF *−857C in the *TNFPA*, *B* and *C* alleles and interacts with the proinflammatory NF-*κ*B subunit transcription factor p65 at the adjacent binding site [114]. Other studies have shown no association between *TNF* gene polymorphisms and severe malaria in Kenya, Malawi, Mali, Tanzania, and Indonesia [111, 115–117]. It has been shown that lymphotoxin-a (LTa), which belongs to the TNF family, plays an important role in malaria [116]. Since LTa binds to the TNF receptors TNF-R1 and TNF-R2, TNF and LTa may exert their effects via the same receptors. *LTA* polymorphisms may influence resistance to malaria in humans, and two SNPs have been described: *LTA* C+80A (rs2239704) and *LTA* A+252G (rs909253). The first is an SNP that allows specific binding of the transcriptional repressor ABF-1 and, therefore, considered to be a low LTa-producing allele, has been associated with lower *P. falciparum* parasitemia in malaria-endemic Burkina Faso but was not associated with severe malaria in Gambia [118–120]. The SNP rs909253 has been reported to influence LTa production [121], but it was not associated with severe malaria in Sri Lanka [113]. Both SNPs (rs2239704 and rs909253) were reported to not be associated with severe malaria in a study from Gambia, Kenya, Malawi, and Indonesia [111, 116].

The chromosomal region 5q31–33 contains several important genes encoding molecules, such as cytokines, growth factors, and growth factor receptors, which are involved in immunity against *Plasmodium* infection [122]. The 5q31–33 region contains genes encoding cytokines IL-3, IL-4, IL-5, IL-9, IL-12B, IL13, and other genes, such as the *immunologically active interferon regulatory factor-1* [123]. Concerning the genetic control of blood infection levels, linkage analyses studies have demonstrated the involvement of the 5q31–q33 region with parasitemia in populations from Cameroon and Burkina Faso [124, 125]. Asymptomatic parasite density was also linked to chromosome 5q31 in a study from Senegal [126]. A genomewide linkage study revealed three strongly suggestive lines of evidence for linkage between mild malaria attacking both the 6p25.1 and the 12q22 regions and between the 20p11q11 region and the prevalence of parasite density in asymptomatic Senegalese children [127]. Furthermore, in this study, one gene associated with malaria infection in the 5q31–q33 was also detected, confirming the importance of this genetic region in the susceptibility to malaria infection [127].

Type 1 helper T lymphocytes may be protective through the release of IFN-*γ*, which activates macrophages to destroy parasitized erythrocytes, promotes the production of opsonizing antibodies, and helps to destroy parasites during the hepatic cycle [128]. However, IFN-*γ* also has proinflammatory effects that may contribute to disease severity [129, 130]. Studies have reported associations between *IFNG* gene polymorphisms and susceptibility to disease. The first intron of the *IFNG* gene contains a highly polymorphic CA-repeat microsatellite, whose 12 CA-repeat allele is associated with high levels of IFN-*γ* production in vitro [131], and it has been associated with an SNP allele *IFNG*+874T (rs62559044), which coincides with a putative NF-*κβ* binding site [132]. In Gambia, no evidence of a strong association between severe malaria and the 12 CA-repeat allele and *IFNG*+874 (rs62559044) polymorphism was observed [133]. However, 14 CA repeats (*IFNG* CA14) were associated with CM in *P. falciparum*-infected children, and *IFNG *−183G/T (no rs designation available) and *IFNG*(CA)14/(CA)14 genotypes were more frequent in children with uncomplicated malaria than in children with cerebral malaria from Mali [134]. 

Concerning *IL-13*, an SNP −1055T>C (rs1800925) has showed a significant association with protection from severe malaria in Thailand [135]. A fine association mapping in the *IL-13* gene using the same malaria subjects revealed that only rs1881457 located in the promoter region, which is in linkage disequilibrium with rs1800925, showed a significant association with severe malaria [123]. Furthermore, two SNPs (rs848, rs1881457) in *IL-13* gene were found to be significantly different between those who have experienced one or more malaria attacks within past 10 years and those who did not in Sri Lanka [136].

IL-12 is a proinflammatory cytokine that boosts erythropoietic responses in infections with *Plasmodium* parasites. Low levels of IL-12 have been associated with the pathogenesis of malaria in children and nonimmune adults through the promotion of IFN-*γ* release from cells of the innate immune system, while high levels of this cytokine are associated with severe malaria [137]. IL-12 cytokine is a dimer composed of a 35-kD subunit encoded by the *IL12A* gene (chromosome 3p12-q13.2) and a 40-kD subunit encoded by the *IL12B* gene (chromosome 5q31–33), which exerts its effects on the immune response through receptors encoded by *IL12RB1* and *IL12RB2* [138]. A mutation in the promoter region of *IL12B*, *IL12B-pro* (rs17860508) has been associated with susceptibility to cerebral malaria [139, 140]. This polymorphism has been shown to affect gene expression and the production of cytokines and nitric oxide (4 bp less) [141]. Moreover, polymorphisms in *IL12A* (rs2243140) and *IL12RB1* (rs429774) confer protection against severe malarial anemia [138].

IL-4 is a pleiotropic cytokine with multiple immune-modulating functions in several cells [142]. IL-4 plays an important role in IgE antibody antimalarial responses and regulates the differentiation of precursor T-helper cells into the Th2 subsets that regulate humoral immunity [122, 143]. Several polymorphisms in the *IL-4* gene have been described, and four polymorphisms were described in the promoter region in association with total IgE production [144–146]. Despite the *IL-4 *−589C>T (rs2243250) influence on IgE levels, there was no association with severe malaria [122, 147]. However, a recent study that assesses the influence of 11 polymorphism in *IL4* gene on predisposition to malaria in Mali found a genetic association between *IL4 *VNTR (rs8179190) and others* IL4 *mutations (−33C/T; rs2243267; rs2243268; rs2243282) with severe disease, supporting the view that *IL4* genetic alterations could be a risk factor for malaria severity [129].

IL-1 is an endogenous pyrogen that plays an important role in the innate immune response of the human host to *Plasmodium* infection [148]. Two different genes (*IL1A* and *IL1B*) encode IL-1, which are located in chromosomal region 2q14, an area that also contains genes for IL-1 receptor types 1 and 2 (*ILR1* and *IL1R2*), the IL-1 receptor antagonist (*IL1RN*), and other homologous genes that have not been well characterized [149]. The rapid induction of IL-1*β* might help control invading malaria parasites through the induction of an acute inflammatory response as part of the first line of defense; however, the overproduction of IL-1*β* might cause severe pathogenic effects [150]. Three different SNPs in the promoter region of the *IL1B* gene (−3737G>A, no rs designation available; −1464G>C, no rs designation available; −511A>G, rs16944) have been associated with IL-1*β* plasma levels [151]. The *IL1B *−511A allele was associated with an increased risk of severe malarial anemia and reduced levels of IL-1*β* [123]. In another study conducted in Gambia, significant associations between variations in *IL1A* +4845G>T (rs17561) and *IL1B* +3954C>T (rs1143634) were associated with symptomatic malaria [148].

IL-10 is an anti-inflammatory Th2-type cytokine produced primarily by monocytes and lymphocytes, and IL-10 exhibits various effects in the regulation of the immune response, including downregulating the expression of the proinflammatory (type 1) immune response [152]. The *IL-10* gene is located on chromosome 1q31-32 within the promoter region and includes the well-defined SNPs *IL-10 *−1082A>G (rs1800870), *IL-10 *−819T>C (rs1800871), and *IL-10 *−592A>C (rs1800872) [153]. The SNP haplotype was associated with susceptibility to severe malarial anemia and functional changes in the plasma concentrations of IL-10, TNF-*α*, and IL-12 [154]. However, other studies have shown no evidence of association between the polymorphisms in the *IL-10 *gene and malaria severity [155]. A study in Gambia showed an association between the haplotype of five SNPs (+4949G, rs3024500/+919C, rs1518110/−627G, rs1800872/−1117C, rs1800896/−3585T, rs1800890) and resistance to cerebral malaria and severe anemia [156].

Several SNPs influence the levels of pro- and anti-inflammatory cytokines in malaria infection and might lead to an imbalance between these molecules that favor increased host susceptibility to *Plasmodium*. Thus, polymorphisms in the immune response might influence host disease tolerance against malaria.

### 3.3. Immunoglobulin Receptors and Nitric Oxide (NO)

Receptors for the Fc fragment of IgG (FcyRs) provide an important link between humoral and cellular immune responses. There are three families of FcyR (I, II, and III). The primary function of FcgRs is the activation of accessory cells against pathogens; thus, FcgRs are essential molecules in the host defense against infection [157]. Among the three classes of FcgR (FcgRI, FcgRII, and FcgRIII), the low-affinity FcgRII class is the most broadly distributed [158]. The *Fc*γ*RIIA* gene contains an important SNP with a G>A substitution in the region responsible for encoding the ligand-binding domain in which histidine (H) replaces arginine (R) at position 131 in the extracellular domain (no rs designation available). Both allotypes bind to human IgG1 and IgG3, but the *Fc*γ*RIIA H131* allotype exhibits higher binding affinity to the IgG2 and IgG3 than the *Fc*γ*RIIA R131* allotype, but none effectively binds to IgG4 [159]. The *Fc*γ*RIIA H131* allotype is the only Fc*γ*R that binds with high affinity to IgG2, and this allele is essential for the phagocytosis of microorganisms opsonized with IgG2 and the clearance of immune complexes containing IgG2 [160, 161]. Furthermore, a protective role for IgG2 in malaria infections has been described, which involves the activation of immune effector cells through Fc*γ*RII [162]. The *RR131* genotype protects against high levels of parasitemia, whereas the *HH131* genotype was associated with susceptibility to severe malaria with high parasite burden [158, 163, 164]. An additional study showed an association between the *FcgRIIA-RR131* genotype and severe malaria [165]. 

NO is a highly diffusible, lipid soluble-free radical that mediates the resistance of host severe malaria and other diseases. The production of NO and the cellular expression of enzyme-inducible nitric oxide synthase (NOS2) are associated with protection against severe forms of malaria [166]. The protective effect of NO against *Plasmodium* reflects parasite killing through reactive nitrogen metabolites and a decrease of endothelial adhesion molecules [167, 168]. In humans, NO is produced through the enzymatic conversion of L-arginine to L-citrulline using three different NO synthases (NOS), and NOS2 is induced through the response to pathogens and proinflammatory cytokines [166]. Several polymorphisms in the *NOS2* gene have been associated with malaria severity. In Gambia, the SNP *NOS2 *−954G> C (no rs designation available) has been associated with resistance to severe malaria [169], whereas in another group of Gambian subjects, short forms of the polymorphic microsatellite (CCTTT) in the *NOS2* transcription start site were associated with fatal malaria [170]. However, in Tanzania, neither *NOS2 *−954G>C polymorphisms nor CCTTT repeats were associated with severe malaria [171]. Another SNP in *NOS2*, −1173C>T (no rs designation available), was associated with protection against cerebral malaria in children in Tanzania and severe anemia in malaria individuals of Kenya [172]. However, no association between *NOS2* polymorphisms and susceptibility to malaria was described [166]. 

The FcgR receptor and NO are important molecules involved in malaria outcomes, and several studies have attempted to associate mutations in these genes with increased susceptibility to develop severe forms of malaria. However, the results are conflicting, and no conclusion has yet been determined.

## 4. Mechanisms of Cytoadherence

One of the peculiar characteristics of *P. falciparum*-mediated malaria is the adhesion of infected erythrocytes to capillary endothelium [173]. This association contributes to the pathology of falciparum malaria because it causes microvascular occlusion and inhibits the immune response against parasites [174, 175]. This adhesion is one of the possible mechanisms underlying the pathogenesis of severe forms of malaria, such as cerebral and placental malaria [176–178]. The adhesion molecules include intercellular adhesion molecule 1 (ICAM-1, CD54), platelet/endothelial cell adhesion molecule1 (PECAM-1, CD31), vascular cell adhesion molecule1 (VCAM-1), thrombospondin, E-selectin, P-selectin, CD36, and chondroitin sulfate A [179]. Another characteristic of the virulent phenotype that contributes to the pathogenesis of *P. falciparum* is its ability to form rosettes, a property in which parasitized erythrocytes bind uninfected erythrocytes to form clumps of these cells [180]. The mechanism responsible for the virulence of the rosettes includes the microvasculature obstruction of the bloodstream in high parasitemia, favoring the invasion of merozoites and immune evasion [181–183]. The process of rosetting is mediated through ligand binding of *P. falciparum* to erythrocyte membrane protein1 (PfEMP1), which is expressed on the membrane of an infected RBC among a variety of uninfected RBC receptors, such as serum components, blood group antigens A and B, glucosaminoglycans, and complement receptor 1 (CR1) [184].

Autopsy studies of patients with fatal cerebral malaria or severe malarial anemia showed the sequestration of erythrocytes infected with *Plasmodium* on brain vascular endothelial cells with the increased expression of adhesion molecules, particularly ICAM-1 [185]. The results of a study in Killifi (Kenya) confirmed this association, showing that the adhesion of infected erythrocytes was highest in cerebral malaria compared with the asymptomatic control group [186]. The ICAM-1 (CD54) is a member of the immunoglobulin super-family, and its role in malaria susceptibility is not limited to an interaction with PfEMP1 [187]. ICAM-1 binds lymphocyte function-associated antigen (LFA)-1, facilitating the movement of leukocytes and active nature killer cells beyond the blood brain barrier during *P. falciparum* infection [188, 189]. Two SNPs have been described in the *ICAM-1* gene, *ICAM-1 Killifi* (rs5491), which results from substitution of lysine for methionine at position 56 of the coding sequence, and a less well-described polymorphism (rs5498, K469E) [187]. The *ICAM-1 Killifi* polymorphism has been associated with both the resistance [190, 191] and susceptibility to severe forms of malaria [192]. However, subsequent studies in Gambia, Thailand, Senegal, Nigeria, and Kenya have reported no significant association between malaria phenotypes and either the ICAM-1Kilifi or the SNP identified in exon 6 (rs5498) [187, 193–197].

Most *P. falciparum* antigens bind to the CD36 molecule, and thus CD36 is considered the most important endothelial receptor for the sequestration of infected erythrocytes [186]. CD36 is an 88-kDa glycoprotein expressed on endothelial cells, macrophages, and dendritic cells, among others. However, in contrast to ICAM-1, this molecule is not expressed on the endothelial cells of brain capillaries [198]. CD36 serves as a receptor for several ligands, including low-density lipoprotein cholesterol (LDL-C), collagen, thrombospodin, and anionic phospholipids and participates in macrophage fusion induced through IL-4 [199]. Mutations in the *CD36 receptor* gene have been associated with protection against or susceptibility to severe forms of malaria. The CD36 deficiency might be induced through the two SNPs in the *CD36* gene (T1264G in exon 10, rs3211938 and G1439C in exon 12, no rs designation available), which encode the truncated proteins that were expressed at high frequency in patients with severe malaria in Gambian, Tanzanian, and Kenyan patients [199–201]. This association was confirmed in a study in India, showing an association of the presence of the mutant allele in heterozygous individuals (1264T>G in exon 10) with protection against severe malaria [202]. A screening of *CD36* gene in malaria patients from Thailand identified two SNPs in the promoter region (−14T>C and −53G>T, no rs designation available) that were associated with protection against cerebral malaria and one microsatellite polymorphism in intron 3 with 12 TG repeats that has been associated with the lower risk of cerebral malaria [203]. Genetic alterations in the *CD36* gene influence the malaria outcome, regardless of different conclusions concerning the polymorphisms identified in this molecule, perhaps reflecting differences among the populations and clinical spectrums of the disease.

Platelet-endothelial cell adhesion molecule 1 (PECAM-1/CD31) is expressed in hematopoietic and endothelial cells. This adhesion molecule was identified as an endothelial receptor for erythrocytes infected with *P. falciparum* [204]. The *PECAM-1* gene is polymorphic, and several polymorphisms have previously been described in the extracellular domain (exons three rs668 L/V, exon 8 rs12953 S/N, rs1131012 exon 12 R/G) and promoter region (GATA-2) [205]. Homozygous individuals with L125V and S563N SNPs in the *CD31* gene were associated with an increased risk for developing cerebral malaria in Thailand [206]. However, in Kenya and Papua New Guinea, no association of the L125V SNP with malaria was observed [205]. Furthermore, an SNP in the *PECAM-1* gene (exon 3 rs668 L/V) was identified as a risk factor for malaria in an endemic region, but this gene exhibited a significant association with protection from disease in a nonendemic region [202]. The mutation in exon 3 of the *CD31* gene might affect the regulation of inflammation because it is present within the first IgG-like domain of the PECAM-1 molecule, which has been associated with hemophilic adhesion and regulates leukocyte transmigration [207, 208]. Thus, despite the influence of genetic alterations in the levels of adhesion molecules, polymorphisms might also alter molecular protein structure and impair the binding affinity of other molecules involved in the immunopathogenesis of malaria.

Complement receptor type 1 (CR1/CD35) is a membrane glycoprotein expressed in various cells, including erythrocytes, monocytes, B and T cells, monocytes, and dendritic cells [209]. CR1 binds with high affinity to C3b and C4b components and plays an important role in the clearance of immune complexes [210]. CR1 also plays a role in opsonization and the control of complement activation [209]. The expression of CR1 on erythrocytes has been related to the formation of rosettes, a phenomenon that results from the adhesion of PfEMP 1 on the surface of infected erythrocytes with a variety of membrane receptors on noninfected erythrocytes [180, 211, 212]. This process contributes to the pathogenesis of severe malaria because it causes the obstruction of cerebral capillaries and increases susceptibility to severe malaria anemia [186, 213]. Furthermore, erythrocyte CR1 binds immune complexes in the bloodstream through a process of “immune adherence” and removes them through phagocyte capitation in the liver and spleen [214]. Subjects with high levels of CR1 on erythrocytes are more likely to form rosettes and contribute to the sequestration of cell clumps in the microvasculature of the brain and other vital organs [180]. Moreover, high levels of CR1 also carry immune complex, which might be recognized by monocytes and endothelial cells to produce proinflammatory mediators [184]. The levels of CR1 on erythrocytes are genetically determined and correlated with the HindIII restriction fragment length polymorphism (RFLP) mutation in the *CR1* gene. Homozygous subjects for the 7.4 kb *HindIII* genomic fragment (the *H* allele) have high levels of CR1 on erythrocytes, whereas homozygous individuals for the 6.9-kb genomic *HindIII* fragment (the *L* allele) exhibit low expression, and *HL* heterozygous individuals show intermediary levels of CR1 in the membrane of erythrocytes [211]. The association of this polymorphism with the susceptibility or resistance to malaria is contradictory. In Gambia and other African populations, a significant association between the *L* allele and protection from severe malaria was not observed [193, 215]. In Thailand, the *LL* genotype was demonstrated as a risk factor for severe malaria [216], and in Papua New Guinea, individuals heterozygous for the *L* allele (*HL*) were correlated with protection against severe malaria [217]. A new polymorphism in the promoter region of the *CR1* gene (rs9429942) was associated with higher levels of CR1 on the surface of RBC and protection against cerebral malaria in Thailand [218]. Different associations between the *CR1* genotype with malaria might be associated with the endemicity of malaria in different regions and an under or overestimation of the actual CR1 levels and interactions between *CR1* and other genetic alterations [184].

## 5. Conclusion

Over the past several years, an increase in the number of scientific publications associated with the genetic predisposition to malaria and severe forms of this disease has been observed. As a result of technological advances, studies of SNPs were exchanged for studies with sophisticated gene sequencing and analyses using advanced molecular biology software. On the basis of the discovery of new functional mutations that alter the expression of several proteins fundamentally implicated in malaria pathogenesis, it is possible to individualize patient care depending on host genotype, as previously demonstrated [219]. However, molecular epidemiology studies should always be interpreted with caution because of the differences in the functionality and frequency of the polymorphisms observed in different populations as a result of diverse evolutionary pressure between different ethnicities. 

**Figure 1:**
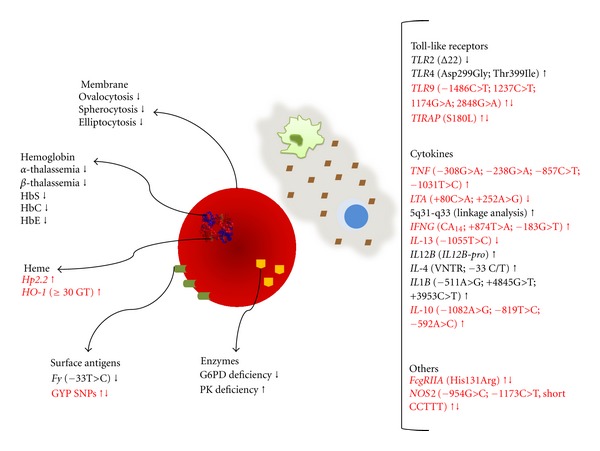
Influence of erythrocyte and immune response gene polymorphisms in malaria outcome. The diagram summarizes the major genetic alterations identified in the erythrocyte and immune response pathways that influence malaria outcome. The up arrow indicates susceptibility, and the down arrow indicates resistance to malaria. Contradictory or not confirmed results are represented by red font color. The protective effect of inherited genetic disorders involving the RBC on malaria infection has been associated with membrane cytoskeleton disorders, surface antigen mutations, enzymatic machinery deficiencies, or hemoglobin and its heme prosthetic group alterations. Considering the immune response, several polymorphisms in gene encoding the TLR receptors and in important cytokines involved in malaria immunopathology are described in the literature. Furthermore, genetic alterations in the FcgRIIA receptor and nitric oxide synthase were also associated with resistance/susceptibility to malaria.

The clinical manifestation of malaria is primarily described by the lysis of infected erythrocytes and subsequent immune and inflammatory response. Thus, it is critical to understand the role of genetic alterations in this pathway that might influence the disease outcome and severity of malaria. Furthermore, it should be observed that not merely one genetic alteration but rather the combination of a set of genetic factors might influence the susceptibility or resistance to malaria (Figure 1). The results from research studies have already shown that individual genetic factors must be considered for the prevention from severe diseases and individual responses to treatment.
